# UroPredict: Machine learning model on real-world data for prediction of kidney cancer recurrence (UroCCR-120)

**DOI:** 10.1038/s41698-024-00532-x

**Published:** 2024-02-23

**Authors:** Gaëlle Margue, Loïc Ferrer, Guillaume Etchepare, Pierre Bigot, Karim Bensalah, Arnaud Mejean, Morgan Roupret, Nicolas Doumerc, Alexandre Ingels, Romain Boissier, Géraldine Pignot, Bastien Parier, Philippe Paparel, Thibaut Waeckel, Thierry Colin, Jean-Christophe Bernhard

**Affiliations:** 1 Bordeaux University Hospital, Urology department, Bordeaux, France; 2 Kidney Cancer group of the French Association of Urology Cancer Committee, Paris, France; 3 SOPHiA GENETICS, Multimodal R&D team, Pessac, France; 4 Angers University hospital, Urology department, Angers, France; 5 Rennes university hospital, Urology department, Rennes, France; 6 HEGP-APHP, Urology department, Paris, France; 7 La Pitié APHP, Urology department, Paris, France; 8 Toulouse university hospital, Urology department, Toulouse, France; 9 Mondor-APHP, Urology department, Paris, France; 10 APHM, Urology department, Marseille, France; 11 IPC, Urology department, Marseille, France; 12 Kremlin-Bicêtre -APHP, Urology department, Paris, France; 13 HCL, Urology department, Lyon, France; 14 Caen University Hospital, Urology department, Caen, France

**Keywords:** Surgical oncology, Risk factors

## Abstract

Renal cell carcinoma (RCC) is most often diagnosed at a localized stage, where surgery is the standard of care. Existing prognostic scores provide moderate predictive performance, leading to challenges in establishing follow-up recommendations after surgery and in selecting patients who could benefit from adjuvant therapy. In this study, we developed a model for individual postoperative disease-free survival (DFS) prediction using machine learning (ML) on real-world prospective data. Using the French kidney cancer research network database, UroCCR, we analyzed a cohort of surgically treated RCC patients. Participating sites were randomly assigned to either the training or testing cohort, and several ML models were trained on the training dataset. The predictive performance of the best ML model was then evaluated on the test dataset and compared with the usual risk scores. In total, 3372 patients were included, with a median follow-up of 30 months. The best results in predicting DFS were achieved using Cox PH models that included 24 variables, resulting in an iAUC of 0.81 [IC95% 0.77–0.85]. The ML model surpassed the predictive performance of the most commonly used risk scores while handling incomplete data in predictors. Lastly, patients were stratified into four prognostic groups with good discrimination (iAUC = 0.79 [IC95% 0.74–0.83]). Our study suggests that applying ML to real-world prospective data from patients undergoing surgery for localized or locally advanced RCC can provide accurate individual DFS prediction, outperforming traditional prognostic scores.

## Introduction

Kidney cancer is showing an increasing incidence worldwide, with 431,288 new cases in 2020^1,2^. It is responsible for a significant mortality rate with almost 180,000 deaths^3^. The growing number of imaging procedures performed each year is leading to an increase in the diagnosis of renal cell carcinoma (RCC) at localized stages for which surgery is the standard of care^4–6^.

The risk of recurrence after surgery is substantial, with rates varying from 20 to 50% at 5 years, depending on the stage^7–9^. In the absence of a consensus, current European recommendations for surveillance are based on prognostic scores that offer only moderate predictive performance^4,10^. These recommendations suggest regular CT scans for 5 to 10 years depending on the patient’s prognostic class.

In this context and as we enter the era of personalized medicine, it becomes increasingly important to accurately predict the individual risk of kidney cancer recurrence after surgery. This would enable the identification of high-risk patients who could be considered for adjuvant treatments, as well as low-risk patients for whom reduced surveillance and radiation exposure are possible.

Machine Learning (ML) analyses large datasets to predict outcomes more accurately than traditional tools. In healthcare, there is a wealth of clinical, biological, pathological, and imaging data available. The exponential growth in information makes analysis and interpretation difficult using traditional statistics, while the ML approach offers promising perspectives and is already being used in many medical specialties^11–13^.

Our aim is, therefore, to use ML on real-world data from a large prospective cohort of patients to propose an individual prediction of the recurrence risk after surgical management of localized or locally advanced kidney cancer.

## Results

### Cohort description

A total of 3372 patients managed for localized or locally advanced RCC were included. The median age was 62 years (IQR 52–69), and 2319 (69%) patients were male. The median tumor size was 4 cm (IQR 2.8–6.2), with a majority (66%) of pT1 and 71% of clear cell RCC (ccRCC). Baseline patient and tumor characteristics are reported in Table 1.

**Table 1:** Patients tumors and characteristics

	Overall, *N* = 3372	Training set, *N* = 2241	Testing set, *N* = 1131
Age (years), median (IQR)	62 (52–69)	61 (51–69)	62 (52–70)
**Sex, n (%)**			
Male	2319 (69)	1541 (69)	778 (69)
Female	1053 (31)	700 (31)	353 (31)
BMI (kg/m^2^), median (IQR)	26.5 (23.7–30.1)	26.3 (23.5–29.7)	27.2 (24.1–30.9)
*Missing*	*97 (3)*	*72 (3)*	*25 (2)*
**ASA score, n (%)**			
1	855 (25)	563 (25)	292 (26)
2	1664 (49)	1161 (52)	503 (44)
≥ 3	574 (17)	381 (17)	193 (17)
*Missing*	*279 (8)*	*136 (6)*	*143 (13)*
**ECOG PS, n (%)**			
0	2150 (64)	1456 (65)	694 (61)
1	483 (14)	347 (15)	136 (12)
≥ 2	131 (4)	82 (4)	49 (4)
*Missing*	*608 (18)*	*356 (16)*	*252 (22)*
**Symptoms at diagnosis, n (%)**			
Asymptomatic	2208 (66)	1472 (66)	736 (66)
Local symptoms	906 (27)	593 (27)	313 (28)
General symptoms	213 (6)	150 (7)	63 (6)
*Missing*	*45 (1)*	*26 (1)*	*19 (2)*
Tumor size (cm), median (IQR)	4.0 (2.8–6.2)	4.0 (2.8–6.5)	4.0 (3.0–6.0)
*Missing*	*18 (0*.*5)*	*12 (0*.*5)*	*6 (0*.*5)*
*Solitary kidney, n (%)*	74 (2)	58 (3)	16 (1)
*Missing*	*28 (0*.*8)*	*21 (0*.*9)*	*7 (0*.*6)*
Bilateral tumors, n (%)	114 (3.8)	82 (4)	32 (3)
*Missing*	*357 (11)*	*250 (11)*	*107 (10)*
**Type of surgery, n (%)**			
Partial nephrectomy	2186 (64.8)	1456 (65)	730 (64.5)
Including RAPN	1521 (45.1)	1089 (48.6)	432 (38.2)
Radical nephrectomy	1186 (35.2)	785 (35)	401 (35.5)
Adrenalectomy, n (%)	440 (13)	301 (14)	139 (13)
*Missing*	*68 (2)*	*39 (2)*	*29 (3)*
Lymphadenectomy, n (%)	202 (6)	144 (7)	58 (5)
*Missing*	*86 (3)*	*49 (2)*	*37 (3)*
NLR, median (IQR)	2.3 (1.7–3.3)	2.2 (1.6–3.2)	2.5 (1.8–3.6)
*Missing*	*943 (28)*	*425 (19)*	*518 (46)*
GFR (ml/min), median (IQR)	86.5 (66.1–111.3)	85.1 (64.8–110.3)	89.0 (68.3–113.0)
*Missing*	*411 (12)*	*180 (8)*	*231 (20)*
**pT, n (%)**			
pT1	2174 (66)	1439 (65)	735 (67)
pT2	257 (8)	157 (7)	100 (9)
pT3a	775 (23)	550 (25)	225 (21)
≥ pT3b	103 (3)	71 (3)	32 (3)
*Missing*	*63 (2)*	*24 (1)*	*39 (3)*
pN + , n (%)	79 (2)	50 (2)	29 (3)
**Fuhrman grade, n (%)**			
1/2	1530 (50)	997 (49)	533 (52)
3	1142 (37)	738 (63)	404 (39)
4	393 (13)	297 (15)	96 (9)
*Missing*	*307 (9)*	*209 (9)*	*98 (9)*
**Histological subtype, n (%)**			
Clear cell	2403 (71)	1568 (70)	835 (74)
Chromophobe	277 (8)	198 (9)	79 (7)
Papillary type 1	337 (10)	245 (11)	92 (8)
Papillary type 2	135 (4)	81 (4)	54 (5)
Others	220 (7)	149 (7)	71 (6)
Positive margins, *n* (%)	130 (4)	72 (3)	58 (5)
Micro vascular emboli on pathology, *n* (%)	446 (15)	313 (15)	133 (13)
Necrotic component on pathology, *n* (%)	1012 (32)	731 (35)	281 (26)
**NSS indication, n (%)**			
Elective	1521 (74)	1026 (72)	495 (78)
Imperative	322 (16)	287 (20)	35 (6)
Relative	220 (11)	113 (8)	107 (17)
*Missing*	*1309 (39)*	*815 (36)*	*494 (44)*

Values in italics represent missing data for each cohort

The median follow up, defined as the median of the intervals between surgery and censoring or death, was 30 months. Four hundred and eighty patients (14.2%) experienced an event over the follow-up (122 locoregional recurrences, 270 metastatic progressions and 88 deaths). The estimated median DFS was 12 years (95% CI 9.2—Inf), and the 5-year DFS probability was estimated at 72.9% (95% CI 70.2–75.5%).

**Fig. 1:**
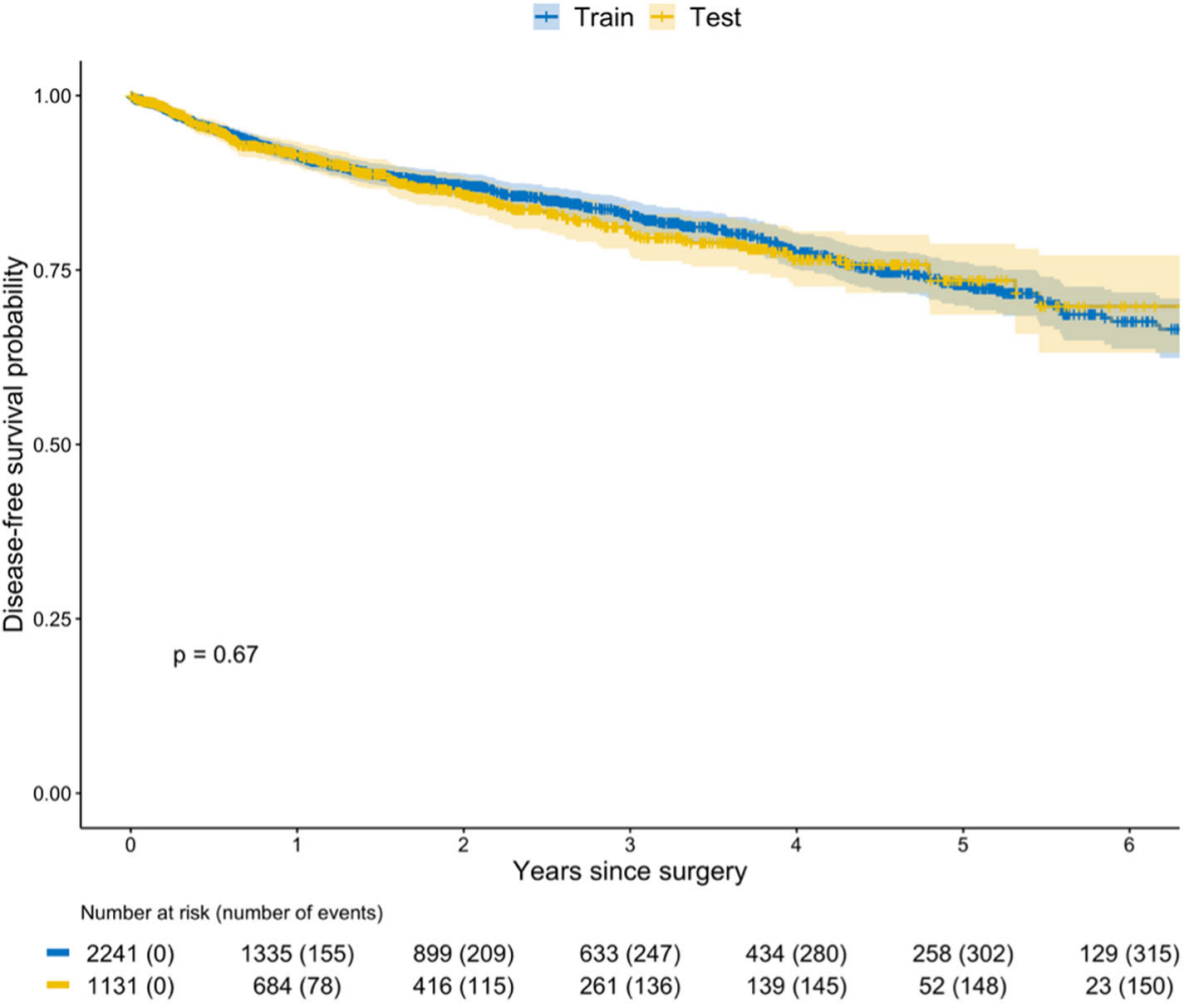
Kaplan–Meier estimates of disease-free survival stratified by train and test datasets. DFS curve of the train cohort (in blue) and of the test cohort (in yellow) were similar (*p* = 0.67).

The training dataset consisted of 2241 patients (66%) from ten centers and 1131 patients (34%) from 13 other centers were assigned to the test dataset. Baseline patient characteristics, missing data rates and DFS curves were similar among the train and test cohorts (Table 1 and Fig. 1).

### Model evaluation

**Fig. 2:**
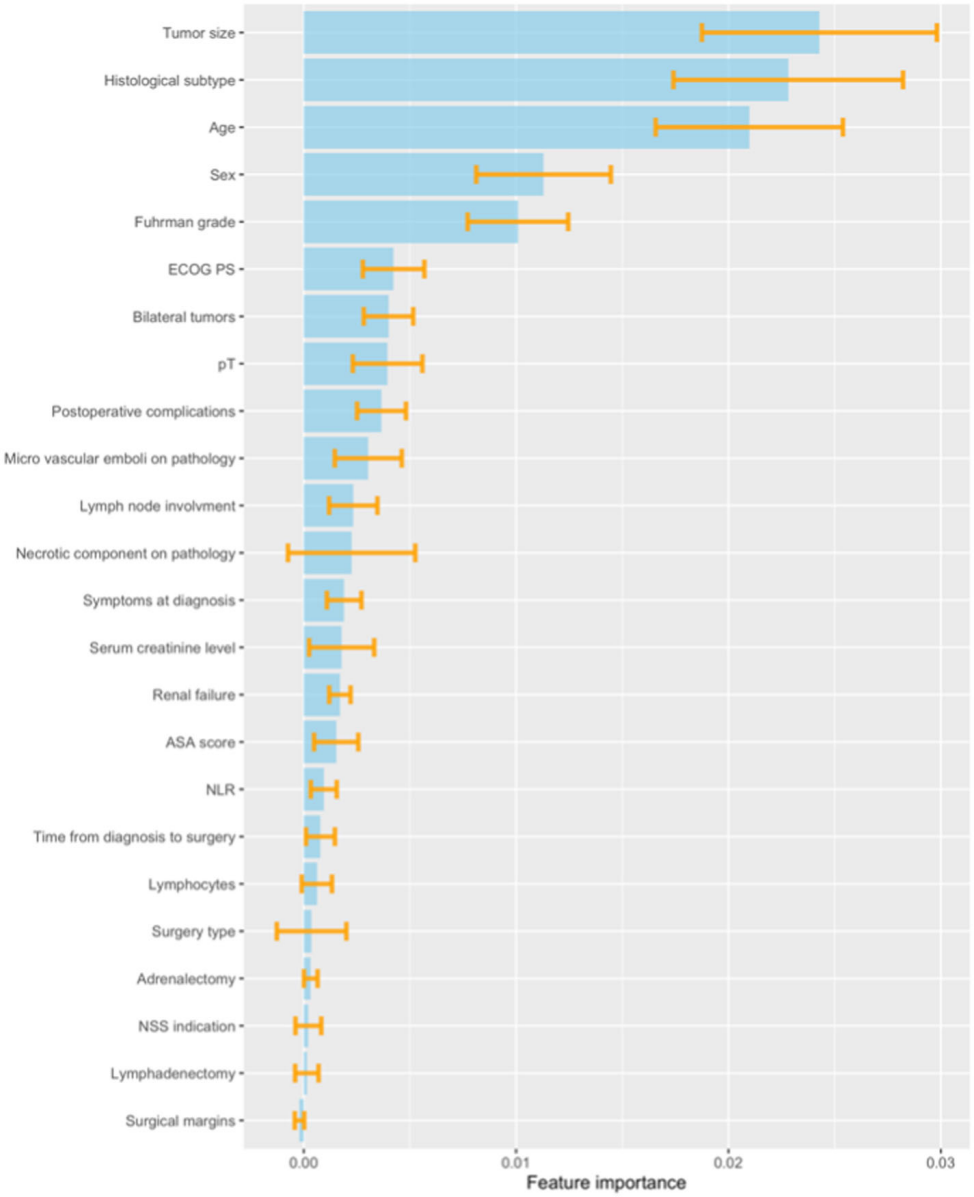
Permutation-based feature importance of the developed ML model. ECOG Eastern Cooperative Oncology Group performance status, NLR Neutrophils to Lymphocytes Ratio; ASA American Society of Anesthesiologists score; NSS Nephron-Sparing Surgery.

The best predictive performance was achieved by combining multiple imputations for missing values and Cox proportional hazards models for time-to-event data. Once features that were not informative due to null variance, redundancy or imbalancement as well as features that were not independently associated with the outcome have been eliminated, the final ML model included 24 clinical, pathological, and biological variables. The permutation-based importance of each feature is displayed in Fig. 2. Tumor size, histological subtype and age at surgery were found to be the most important features of the ML model.

This ML model demonstrated good discrimination and calibration abilities when applied to test dataset, with an integrated AUC of 0.81 [95% CI 0.77–0.85] and an integrated Brier score of 0.11 [0.10–0.13] (Table 2). The robustness of the signature was verified, with stable estimated predictive metrics between the training stage and the external validation stage. Both calibration and discrimination performance decreased over time, certainly due to the decrease over time in both the number of patients still at risk and the number of events observed.

**Table 2:** Predictive performance of the developed ML model

Predictive metrics	Train dataset (cross-validation; *n* = 2241)	Test dataset (external validation; *n* = 1131)
Integrated AUC (0.5, 5 years) [95% CI]	0.81 [0.77–0.84]	0.81 [0.77–0.85]
AUC (*t* = 0.5 yr) [95% CI]	0.85 [0.81–0.89]	0.86 [0.80–0.91]
AUC (*t* = 1 yr) [95% CI]	0.83 [0.79–0.87]	0.86 [0.82–0.90]
AUC (*t* = 2 yr) [95% CI]	0.81 [0.76–0.86]	0.81 [0.76–0.85]
AUC (*t* = 5 yr) [95% CI]	0.77 [0.72–0.82]	0.71 [0.61–0.80]
Integrated Brier score (0.5, 5 years) [95% CI]	0.11 [0.09–0.12]	0.11 [0.10–0.13]
Brier score (*t* = 0.5 yr) [95% CI]	0.04 [0.03–0.05]	0.04 [0.03–0.05]
Brier score (*t* = 1 yr) [95% CI]	0.07 [0.05–0.08]	0.06 [0.05–0.07]
Brier score (*t* = 2 yr) [95% CI]	0.09 [0.07–0.10]	0.10 [0.08–0.12]
Brier score (*t* = 5 yr) [95% CI]	0.16 [0.14–0.18]	0.18 [0.14–0.22]

**Fig. 3:**
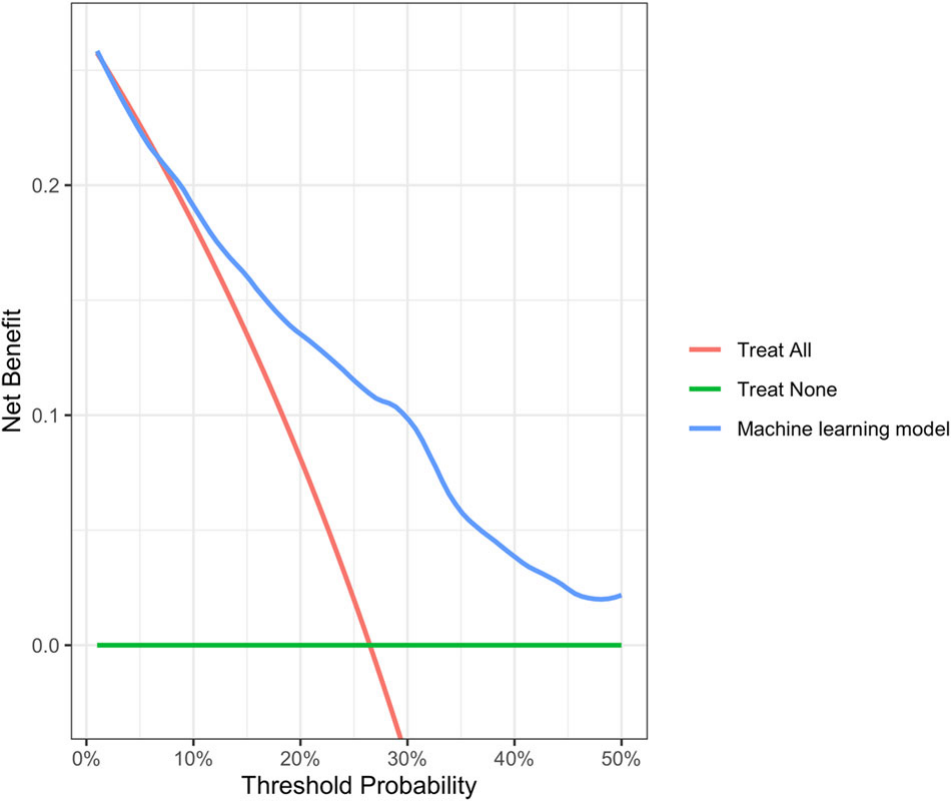
Decision curve. Decision curve for prediction of recurrence risk within 5 years after surgery. The green curve assumes no patient will recur. The red curve assumes all patients will recur. The blue curve is associated with the use of machine learning model. The graph shows the expected net benefit for a range of threshold probabilities. The expected net benefit corresponds to the number of patients for every 100 patients who were correctly predicted with recurrence, without increasing the number of false positive predictions. The machine learning model showed better net benefit than the competing decisions for all the plausible threshold probabilities, comprised between 10% and 50%.

Decision curve analysis (Fig. 3) highlights the clinical utility of using the ML model to predict the recurrence risk within 5 years following surgery, with higher net benefit for the ML model than the competing decisions, assuming that all patients or no patient will recur, for all threshold probabilities between 10% and 50%. For a threshold probability of 30%, the ML model achieved a net benefit of 0.10, which means that 10 additional recurrences for every 100 patients would have been identified, without increasing the number of false positive predictions.

### Individual prediction and stratification into risk groups

**Fig. 4:**
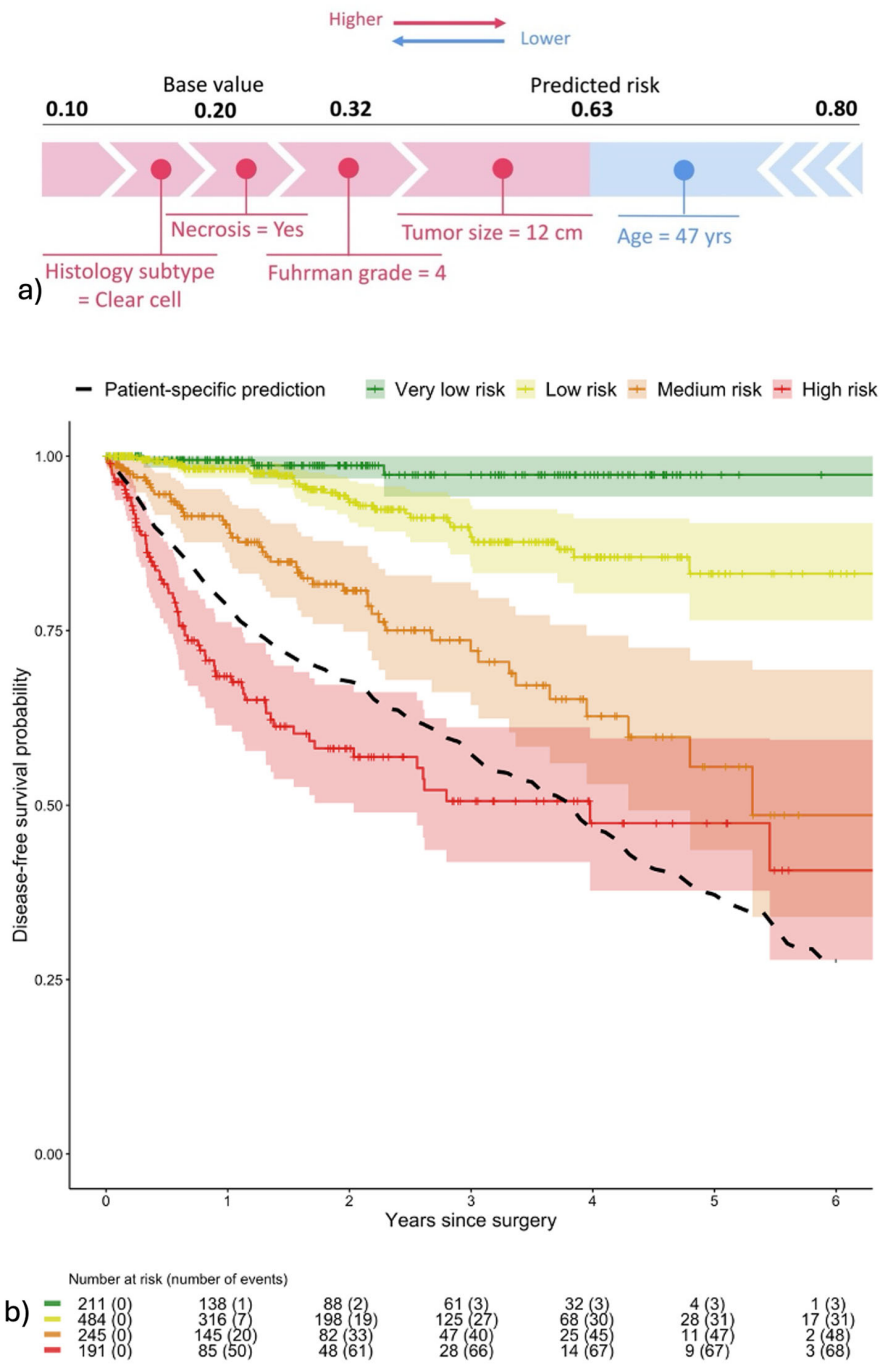
Interpretability tools. **a** SHAP value. Individual risk of recurrence within five years after surgery explained using SHAP values for a patient. The average estimated risk in the train population (base value) is 20%. Individual risk prediction for the patient is higher, at 63%, with features in red that increase the patient’s risk of recurrence and features in blue that decrease it. **b** Risk groups' stratification. Actual disease-free survival in the test cohort (*n* = 1131) according to the stratified risk score. 211 (18.7%) were classified as very low risk, 484 (42.8%) patients at low risk, 245 (21.7%) patients at medium risk and 191 (16.9%) patients at high risk of recurrence within 5 years following the surgery. The black curve represents the predicted survival curve for the patient in (**a**).

Each individual prediction was displayed using SHAP values, with characteristics that increase the risk of recurrence in red and protective factors in blue. An example is given in Fig. 4a, in which the patient has a 5-year recurrence risk of 63% (compared with an average risk of 20% in the training population). This increased risk is explained by the clear cell histological subtype, the Fuhrman grade 4, the presence of a necrotic component and the large tumor size. The young age of the patient reduces this risk. Patients assigned to the test cohort were stratified into four risk groups (Fig. 4b) using thresholds determined from the train cohort, achieving an iAUC of 0.79 [IC95% 0.74–0.83]. The threshold for the very low-risk group was set to include patients with a recurrence risk within 5 years lower than 10%. The resulting group represents 19% of the population with an actual 5-year recurrence rate lower than 2% and no death observed within this time frame. The threshold for low and medium-risk patients was set to obtain recurrence risks within 5 years between 10% and 22% and between 22% and 41%, respectively. This represents 43% of the population with an actual 5-year DFS of 83% for the low-risk group and 22% of the population with a DFS of 54% for the medium-risk group. Finally, the last group isolates patients with a recurrence risk superior to 41% within 5 years resulting in 17% of the population having an actual 5-year DFS of 49%.

### Comparison of predictive performance

The performance of the ML model was compared with conventional risk scores on the test dataset (Table 3). The UISS, SSIGN, GRANT and Leibovitch risk scores could be calculated for 882 (78%), 946 (84%), 1008 (89%) and 578 (51%) patients, respectively, due to incomplete data. The machine learning model outperformed the GRANT (*p* < 0.001), SSIGN (*p* = 0.01), and UISS (*p* < 0.001) risk scores. Additionally, it was available for twice as many patients as the Leibovich-2018.

**Table 3:** Comparison of the predictive performance of the models in terms of calibration and discrimination

Risk score	N (%)	Integrated AUC (0.5, 5 years) [95% CI]			Brier score (5 years) [95% CI]		
		Prognostic score	ML model	*p*-value	Prognostic score	ML model	*p*-value
ML model	1131 (100)	–	0.81 [0.77, 0.85]	–	–	0.18 [0.14–0.22]	–
GRANT	1008 (89)	0.65 [0.61–0.70]	0.82 [0.77, 0.86]	<0.01	0.20 [0.15–0.26]	0.13 [0.11–0.16]	<0.01
SSIGN	946 (84)	0.78 [0.73–0.83]	0.81 [0.76, 0.85]	0.01	ND	ND	ND
UISS	882 (78)	0.70 [0.65–0.75]	0.82 [0.77–0.86]	<0.01	0.15 [0.13–0.18]	0.13 [0.10–0.16]	< 0.01
Leibovich 2018	578 (51)	0.76 [0.67–0.83]	0.78 [0.70–0.85]	0.17	0.13 [0.10–0.17]	0.13 [0.09–0.16]	0.20

Calibration for SSIGN could not be computed on DFS outcome as estimated risks are only provided for cancer-specific survival (CSS).

## Discussion

According to the literature, 20–50%^7^ of patients with localized or locally advanced kidney cancer will develop recurrence after surgery. Accurate and routinely usable predictive models of this risk are, therefore essential to advise patients and set up follow-up or propose adjuvant treatment. We have developed a predictive model of DFS after surgery in a multicenter NCI-HAS co-labelled cohort of patients with localized or locally advanced kidney cancer. We used clinical, biological, and pathological data available in routine practice.

The variables that were found to be of utmost importance in our predictive model are consistent with known prognostic factors and have been used in several prognostic models. Indeed, the tumor, node, and metastasis (TNM)^14^ classification has been one of the most used prognostic factors for years. The same applies to Fuhrman grade^15^ and histological subtype, which are recommended by the EAU guidelines^4^. Several studies have shown that patients with ccRCC have a worse prognosis than those with papillary and chromophobe RCC^16,17^. Performance status is recognized as an important predictor of clinical outcomes and is a common inclusion criterion in clinical trials. Finally, a meta-analysis including almost 15,000 patients showed a 2-to-3-fold higher risk of recurrence, metastatic progression, and cancer-related death in patients with vascular emboli on pathology^18^.

The association of inflammatory markers with poor prognosis has been demonstrated in several cancers^19^ and the neutrophil-to-lymphocyte ratio (NLR) is often used as a prognostic biomarker^20^. In kidney cancer, its predictive value has been evaluated several times^21–23^.

The UISS^24^, developed on a retrospective cohort of 661 patients, classifies patients with localized kidney cancer into 3 risk groups based on Fuhrman grade, ECOG score and pT stage. Its predictive value is moderate with a c-index ranging between 0.56 and 0.72 in different external validation studies^25–27^. The SSIGN system, which integrates stage, tumor size, Fuhrman grade and the presence of a necrotic component, predicts cancer-specific survival (CSS) in patients with ccRCC, with a c-index of 0.84 in the initial cohort. However, the accuracy is somewhat lower in different external validation studies, with c-indexes ranging between 0.63 and 0.78^26^. Leibovich et al. developed three different models depending on the histological type of the patient (clear cell, papillary or chromophobe). They used a CoxPH model on a monocentric cohort, with c-indexes for DFS and CSS of 0.83 and 0.86, respectively. Once again, the performance seems to be slightly lower in external validation studies (c index ranging from 0.73 to 0.81^25,28^). Finally, the GRANT score has been recently published. It includes Fuhrman grade, age, stage, and lymph node involvement, classifying patients into two risk groups. Its external validation revealed a low concordance score of 0.59^29^.

As the predictive performance of these models appears to be moderate, a few articles have suggested using machine learning to predict recurrence with greater accuracy. Therefore, Buyn et al. ^30^ developed a model to predict recurrence-free survival (RFS) and CSS in a cohort of 2139 ccRCC patients. The best results were obtained with a DeepSurv model. Meanwhile, Kim et al. ^31^ described a high accuracy in predicting recurrence using a Naive Bayes model in a cohort of 2814 patients. Nevertheless, the methodology used to develop and validate these models is poorly described and no individual predictions are presented in these articles.

More recently, Khene et al. ^25^ published a model based on a cohort of 4067 patients randomly assigned to either a training or a test group. They tested three machine learning algorithms and found that the Random Survival Forests model achieved the highest c-index (0.79). However, the paper had some methodological limitations and statistical biases. It lacked external center validation, did not investigate risk group stratification, had unclear handling of missing data, and did not address the applicability of usual risk scores in cases of incomplete observations. Patients included in this study were also enrolled in the UroCCR database, which may lead to minimal patient overlap between the two studies. However, considering that the UroCCR database comprises over 16,500 patients from 44 different centers and that the methodologies of our two studies differ, the cohorts and findings of our studies are distinct. This contributes new evidence to the prediction of recurrence in localized or locally advanced kidney cancer.

Furthermore, Gui et al. ^32^ published a multimodal model that combines genomic and pathomic with clinical features to predict the recurrence-free interval after surgery in a cohort of patients with ccRCC, using a nomogram. Nevertheless, there are several limitations, beginning with data obtained from a retrospective review of clinical files, in contrast to our data obtained from a real-world database collected prospectively. Clinical data are also selected a priori and based on the outdated Leibovich 2003 score^33^, which was revised in 2018. Additionally, the utilization of pathomics and genomics remains in the realm of research. These technologies are indeed prohibitively expensive and not readily available for routine clinical use. The authors themselves acknowledge that the associated tasks are too time-consuming for large-scale clinical application.

Recently, results from the Keynote 564 trial^34^ were reported, showing for the first time a benefit of adjuvant immunotherapy on DFS in patients who underwent surgical management for localized kidney cancer. However, with a median follow-up of 30 months, approximately 60% of patients in the placebo group remained disease free, while about 19% of patients in the experimental group experienced grade 3–5 adverse events. Therefore, it is essential to identify the right candidates for such treatment, specifically patients whose risk of recurrence justifies the use of a drug with potentially significant and long-lasting side effects. Furthermore, other phase III adjuvant trials have failed to demonstrate any post-surgery benefits^35,36^, and the selection of patients deemed high risk is a matter of controversy. Patients with relatively low recurrence risk may have been included, potentially masking the improvements in clinical outcomes offered by adjuvant therapy. Utilizing ML algorithms could enhance patient screening and the selection of patient profiles that would derive greater benefits from adjuvant treatment.

Our model provides individual DFS prediction following surgery for localized RCC with a high degree of accuracy. It outperformed most of the common prognostic scoring systems and offers the advantage of predicting outcomes for every single patient, even with incomplete data, in contrast to traditional scores. Displaying the SHAP values allows to explain the prediction and the impact of each factor at the individual patient level. Integrating this tool into the UroCCR database will automatically provide physicians with the individual risk assessment for each patient included, enabling personalized management and follow-up. The prediction algorithm will also be publicly available on the website of the French kidney cancer research network (www.uroccr.fr). The model variables will then have to be entered manually.

Additionally, our model can be used to stratify patients into four distinct prognostic categories with strong discriminatory power. This allows us to identify a group of patients with a very low risk of recurrence, constituting 19% of the overall cohort. In this population, a less intensive post-operative follow-up can be considered, thus reducing medical costs and radiation exposure.

While the strengths of our study include a large number of patients who are representative of the population managed for localized or locally advanced kidney cancer, and a method for external validation of the model that allows for its generalization, it also has some limitations. First, the study is retrospective, secondly the median follow-up time of 30 months is relatively short. We should also mention that ethnicity distribution is not available, as research in France is strictly regulated by the CNIL (French National Commission for Information Technology and Civil Liberties), which prohibits any ethnic categorization. This model is therefore probably not generalizable to African and Asian populations, which are poorly represented in France. Finally, the data are extracted from a multicenter database. Management and follow-up may therefore vary from one center to another. Although most cases were monitored, we can also question the disparity in database completion, particularly in event reporting, which could lead to a potential bias when designing the model.

Finally, as previously mentioned, models perform differently when validated on different cohorts. The same likely applies to our model, which should therefore be validated on other populations and prospectively.

Our study suggests that machine learning applied to real-world evidence dataset from patients undergoing surgery for localized or locally advanced kidney cancer can provide a more accurate individual prediction of DFS compared to conventional prognostic scores. This has the potential to enhance candidate selection for adjuvant therapy and identify patients who would benefit from less intensive surveillance.

## Methods

### Study population

From the French research network on kidney cancer database UroCCR (NCT 03293563), which has been labelled by the French National Cancer Institute (NCI) and the French High Authority of Health (HAS), we included all patients who underwent surgery between May 2000 and January 2020 for a localized or locally advanced renal cell carcinoma (pTany, Nany, M0). Patients with hereditary RCC, non-primary renal tumors, benign lesions, concomitant malignant disease or metastases and patients without any news after surgery or insufficient data were excluded. The surgical procedure could have been partial or radical nephrectomies, performed either via laparoscopic or open approach in one of the 23 participating tertiary centers. To utilize biological data, we chose to exclude patients with pathologies that could modify blood tests (hemopathies, chronic inflammatory diseases) (Supplementary Fig. 1). All data were collected prospectively in the UroCCR database after obtaining written consent. It was approved by the French Advisory Committee on the Processing of Health Research Information and the French Data Protection Agency, and it complies with all ethical regulations including the Declaration of Helsinki.

### Study objectives

The primary objective was to predict individual disease-free survival (DFS) based on baseline multimodal data. The secondary objective was to stratify patients into risk groups to identify a population with very low risk and a population at high risk of recurrence within 5 years following surgery.

### Predictors

We extracted more than 200 demographic and clinical variables, including sex, age at surgery, American Society of Anesthesiologists (ASA) score, body mass index (BMI), Eastern Cooperative Oncology Group performance status (ECOG PS), symptoms at diagnosis, chronic kidney disease (CKD) score and time from diagnosis to surgery. Biological data included hemoglobin, thrombocytes, leucocytes, polymorphonuclear neutrophils (PMN), lymphocytes and serum creatinine level. Preoperative tumor characteristics encompassed size on contrast-enhanced imaging and multifocal or bilateral status. Surgical data collected comprised duration, nephrectomy type (partial vs. total), approach (laparoscopic vs. open), blood loss, presence of lymph node dissection or adrenalectomy as well as intra and postoperative complications.

Finally, we examined pathological findings including tumor size and stage, Fuhrman grade, histological subtype, surgical margins, and the presence of necrosis or microvascular invasion.

### Follow-up and outcome

Post-operative follow-up was conducted according to common practices of each center, typically aligning with the recommendations of the French Society of Urology^37^, including visits at post-operative month 1–3 and every 6 months for 3 years, followed by annual visits. Radiological follow-up involved a contrast-enhanced examination of the abdomen and pelvis (CT scan or MRI) and a chest CT scan.

The primary outcome was DFS, defined as the time elapsed between surgery and the diagnosis of local recurrence, metastatic progression, or death from any cause, whichever occurred first.

### Hold-out validation

**Fig. 5:**
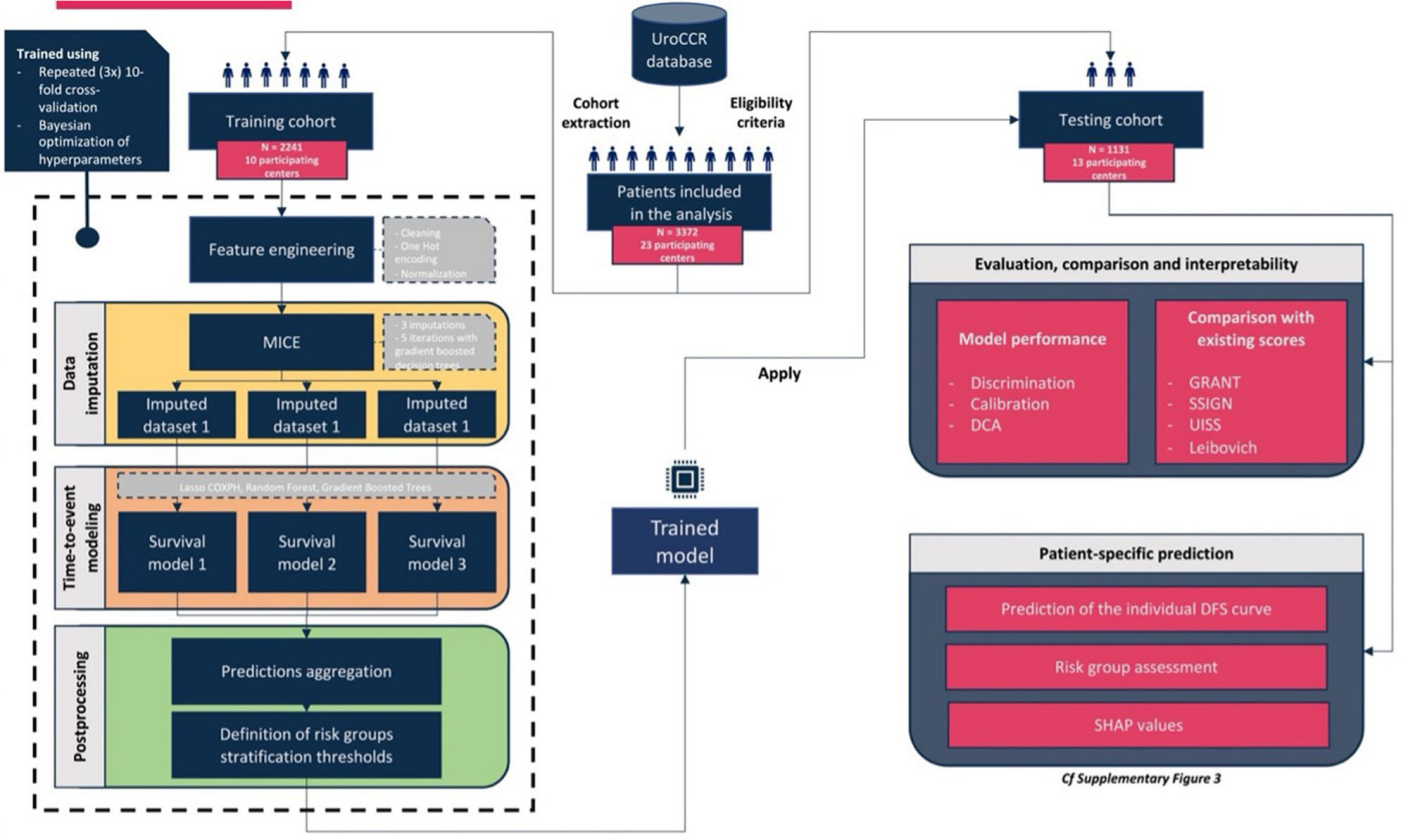
Workflow for machine learning model (ML) development and evaluation. Two thousand two hundred and forty-one patients from 10 centers were randomly assigned to the training cohort. Missing data were multiply imputed and several time-to-event models were trained. The best trained model was then externally validated on the testing cohort of 1131 patients from 13 different centers and compared with existing risk scores.

Participating sites were randomly assigned to either the training or testing cohort, ensuring an approximate 2:1 ratio of patients and similar distributions of DFS. The model and risk groups thresholds were optimized on the training cohort and then applied to the testing cohort to evaluate predictive performance. The workflow for machine learning model development and evaluation is illustrated in Fig. 5.

### Model development

Categorical features with unbalanced modalities were recoded. Categorical features were then one-hot encoded while numerical features were normalized. Missing data were multiply imputed (3 imputations) using the MICE (Multiple Imputation using 5 Chained Equations) algorithm^38,39^ and gradient-boosted decision trees. Several time-to-event models were trained on the training data set including Cox Proportional Hazards models with LASSO regularization^40^, random survival forests and gradient-boosted survival trees. Hyperparameters of each algorithm (Table 4) were tuned using repeated cross-validation procedure (3 × 10 folds) and Bayesian optimization of the integrated AUC (iAUC) over the time window (6, 60 months after surgery). Table 4 lists the explored spaces of each hyperparameter. The discriminative power of the machine learning (ML) models was assessed using iAUC, which represents the averaged cumulative-dynamic time-dependent area under the ROC curve (AUC) over the studied time interval. The iAUC values range from 0 to 1, with 0.5 for a random prediction and 1 for a perfect discrimination ability. Blanche et al. ^41^ argued that the AUC should be preferred to the C-index^42^ because the former compares the ranks of the predictions with the binary event status while the latter compares the ranks of the predictions with the ranks of the actual event status.

**Table 4:** Models’ parameters space for Bayesian optimization

Model	Parameter	Parameters space		Optimal value
		Min value	Max value	
COXPH	Alpha	0.000001	0.1	0.004852664490837273
	L1 ratio	0.01	1	0.2422240393513387
Random Forest	Number of estimators	50	300	109
	Min samples split	20	100	24
Gradient Boosting	Number of estimators	10	100	100
	Learning rate	0.01	1	0.342195080906687
	Min samples split	5	40	40

### Model evaluation

The ML model with the best cross-validated predictive performance was chosen and evaluated on the test dataset for external validation.

The model was assessed in terms of both discrimination and calibration using the time-dependent AUC and the time-dependent Brier score. The Brier score is used to measure the model calibration, ranging from zero to one with zero being the best score and one the worst. The metrics were estimated using Kaplan–Meier-based inverse probability censoring weighting (IPCW) to consider censoring. 95% confidence intervals were calculated, using Nadeau and Bengio correction^43^ for the cross-validation stage on the training dataset, and percentile bootstrapping for the external validation on the test dataset.

The clinical utility of the model was assessed using a decision curve based on the estimated risks of recurrence within five years following surgery. The permutation-based importance of each feature in the whole ML model was evaluated by computing the decrease in the optimization metric when the values of a given feature are randomly shuffled. SHAP (SHapley Additive exPlanations) values^44^ were then computed to explain each patient’s predicted probability of recurrence within the 5 years following surgery.

### Stratification into risk groups

**Fig. 6:**
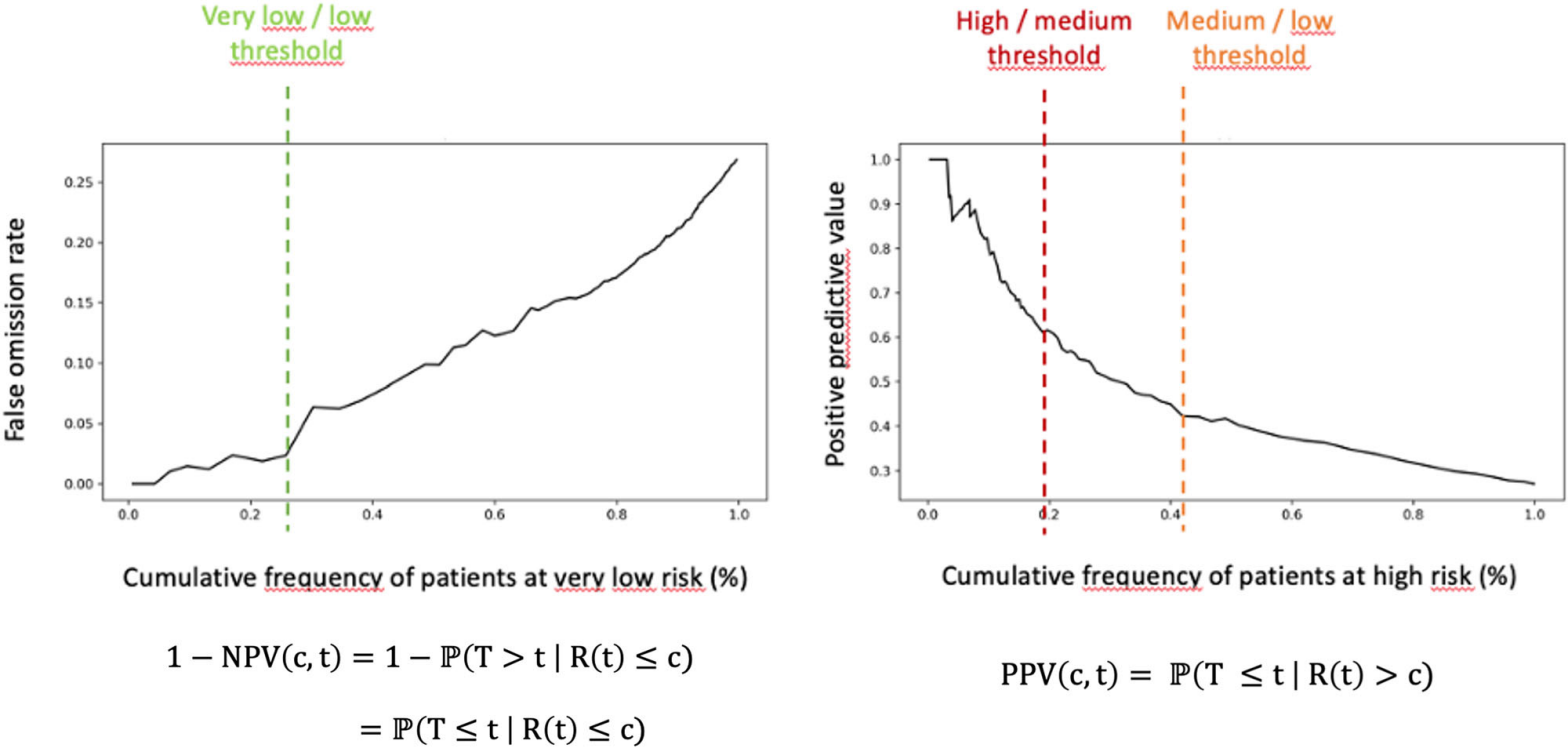
Negative predictive value and positive predictive value at 5 years on the training cohort. Determination of the stratification thresholds on the training cohort. The left-side Figure shows the false omission rate (equivalent to 1—Negative Predictive Value) at five years according to various decision thresholds. The right-side Figure shows the positive predictive value at five years according to various decision thresholds. The machine learning model provides a relapse risk for all horizon times t that have been seen in the training dataset. For our use case, we decided to set t to 5 years as it is the standard horizon clinicians would consider building surveillance plan for their patients. Our primary goal is to find a significant group of patients with a very low risk of recurrence at 5 years. To do so, we decided to plot the false omission rate as a function of the cumulative frequency of patients in the very low-risk group by varying the risk threshold. We define our very low risk threshold such as there is a significant increase in the false omission rate. We can then use a similar strategy with the positive predictive value (PPV) to determine a high-risk group of patients. We look for PPV “plateau” to determine the risk thresholds. This method is reused to differentiate medium and low-risk groups.

Patients were stratified into four risk groups of recurrence within five years following surgery: very low, low, medium, and high-risk groups. The thresholds were notably set to obtain a large proportion of patients with a very low actual relapse rate (very low-risk group) and a significant proportion of patients with a high actual relapse rate (high-risk group). These stratification thresholds were determined using the training dataset and then applied to patients in the test dataset (Fig. 6). The DFS curves for the four risk groups were estimated using the Kaplan–Meier method and compared using log-rank tests.

### Comparison with usual risk scores

The ML model was compared to four prognostic scores commonly used in guidelines and clinical trials: the UISS (University of California at Los Angeles Integrated Staging System)^24^, the SSIGN (Stage, Size, Grade, and Necrosis)^45^, the GRANT (GRade, Age, Nodes and Tumor)^46^ and, the Leibovich^47^ scores. These prognostic scores could not be computed for the entire testing cohort due to incomplete observations. Each pairwise comparison was conducted on the subset of patients for whom the prognostic score was available, with one-sided *p*-values estimated using bootstrapping.

This model has been developed in accordance with the SPIRIT-AI guidelines^48^, and its integration does not require any specific requirements. The data and algorithm can be made available upon request.

### Reporting summary

Further information on research design is available in the Nature Research Reporting Summary linked to this article.

### Supplementary information

Supplementary figure 1

REPORTING SUMMARY

## Data Availability

Requests for specific data will be considered by the UroCCR scientific committee immediately following the publication of the manuscript for researchers who provide a methodologically sound proposal. Data include access to the deidentified participant data collected during the study. The request should be sent to uroccr@chu-bordeaux.fr. To gain access, data requestors will sign an NDA.

## Code availability

Requests for specific information regarding code or algorithm construction will be provided on demand by the UroCCR team. Table 4 lists the explored spaces of each hyperparameter of the algorithm. The algorithm will be publicly available on the UroCCR website (www.uroccr.fr).

## References

[1] Bukavina L (2022). Epidemiology of renal cell carcinoma: 2022 update. Eur. Urol..

[2] Sung H (2021). Global Cancer Statistics 2020: GLOBOCAN estimates of incidence and mortality worldwide for 36 cancers in 185 countries. CA Cancer J. Clin..

[3] Ferlay J (2018). Cancer incidence and mortality patterns in Europe: Estimates for 40 countries and 25 major cancers in 2018. Eur. J. Cancer.

[4] Ljungberg B (2022). European association of urology guidelines on renal cell carcinoma: the 2022 update. Eur. Urol..

[5] Kane CJ, Mallin K, Ritchey J, Cooperberg MR, Carroll PR (2008). Renal cell cancer stage migration: analysis of the National Cancer Data Base. Cancer.

[6] Turner RM, Morgan TM, Jacobs BL (2017). Epidemiology of the small renal mass and the treatment disconnect phenomenon. Urologic Clin. North Am..

[7] Williamson TJ, Pearson JR, Ischia J, Bolton DM, Lawrentschuk N (2016). Guideline of guidelines: follow-up after nephrectomy for renal cell carcinoma. BJU Int..

[8] Jamil ML (2020). Long-term risk of recurrence in surgically treated renal cell carcinoma: a Post Hoc Analysis of the Eastern Cooperative Oncology Group—American College of Radiology Imaging Network E2805 Trial Cohort. Eur. Urol..

[9] Borregales LD (2016). Prognosticators and outcomes of patients with renal cell carcinoma and adjacent organ invasion treated with radical nephrectomy. Urol. Oncol..

[10] Escudier B (2019). Renal cell carcinoma: ESMO Clinical Practice Guidelines for diagnosis, treatment and follow-up. Ann. Oncol..

[11] Bora A (2021). Predicting the risk of developing diabetic retinopathy using deep learning. Lancet Digital Health.

[12] Tran KA (2021). Deep learning in cancer diagnosis, prognosis and treatment selection. Genome Med..

[13] Boulenger de Hauteclocque, A. et al. Machine-learning approach for prediction of pT3a upstaging and outcomes of localized renal cell carcinoma (UroCCR-15). *BJU Int.*10.1111/bju.15959 (2023) 10.1111/bju.15959 36648124

[14] Compérat E (2019). Comparison of UICC and AJCC 8th edition TNM classifications in uropathology. Ann. Pathol.

[15] Fuhrman SA, Lasky LC, Limas C (1982). Prognostic significance of morphologic parameters in renal cell carcinoma. Am. J. Surg. Pathol..

[16] Cheville JC, Lohse CM, Zincke H, Weaver AL, Blute ML (2003). Comparisons of outcome and prognostic features among histologic subtypes of renal cell carcinoma. Am. J. Surg. Pathol..

[17] Patard J-J (2005). Prognostic value of histologic subtypes in renal cell carcinoma: a multicenter experience. J. Clin. Oncol..

[18] Huang H (2015). Microvascular invasion as a prognostic indicator in renal cell carcinoma: a systematic review and meta-analysis. Int. J. Clin. Exp. Med..

[19] Grivennikov SI, Greten FR, Karin M (2010). Immunity, inflammation, and cancer. Cell.

[20] Templeton AJ (2014). Prognostic role of neutrophil-to-lymphocyte ratio in solid tumors: a systematic review and meta-analysis. J. Natl Cancer Inst..

[21] Nunno VD (2019). Prognostic impact of neutrophil-to-lymphocyte ratio in renal cell carcinoma: a systematic review and meta-analysis. Immunotherapy.

[22] Allenet C (2022). Can pre-operative neutrophil-to-lymphocyte ratio (NLR) help predict non-metastatic renal carcinoma recurrence after nephrectomy? (UroCCR-61 Study). Cancers (Basel).

[23] Pichler M (2013). Validation of the pre-treatment neutrophil–lymphocyte ratio as a prognostic factor in a large European cohort of renal cell carcinoma patients. Br. J. Cancer.

[24] Zisman A (2001). Improved prognostication of renal cell carcinoma using an integrated staging system. JCO.

[25] Khene, Z.-E. et al. Application of machine learning models to predict recurrence after surgical resection of nonmetastatic renal cell carcinoma. *European Urol. Oncol.* S2588931122001377 10.1016/j.euo.2022.07.007 (2022) 10.1016/j.euo.2022.07.007 35987730

[26] Usher-Smith JA (2022). Risk models for recurrence and survival after kidney cancer: a systematic review. BJU Int..

[27] Correa AF (2021). Predicting disease recurrence, early progression, and overall survival following surgical resection for high-risk localized and locally advanced renal cell carcinoma. Eur. Urol..

[28] Lee HJ, Lee A, Huang HH, Lau WKO (2019). External validation of the updated Leibovich prognostic models for clear cell and papillary renal cell carcinoma in an Asian population. Urol. Oncol..

[29] Khene Z-E (2021). External validation of the ASSURE model for predicting oncological outcomes after resection of high-risk renal cell carcinoma (RESCUE Study: UroCCR 88). Eur. Urol. Open Sci..

[30] Byun S-S (2021). Deep learning based prediction of prognosis in nonmetastatic clear cell renal cell carcinoma. Sci. Rep..

[31] Kim H, Lee SJ, Park SJ, Choi IY, Hong S-H (2021). Machine learning approach to predict the probability of recurrence of renal cell carcinoma after surgery: prediction model development study. JMIR Med Inf..

[32] Gui C-P (2023). Multimodal recurrence scoring system for prediction of clear cell renal cell carcinoma outcome: a discovery and validation study. Lancet Digit Health.

[33] Leibovich BC (2003). Prediction of progression after radical nephrectomy for patients with clear cell renal cell carcinoma: a stratification tool for prospective clinical trials. Cancer.

[34] Choueiri TK (2021). Adjuvant pembrolizumab after nephrectomy in renal-cell carcinoma. N. Engl. J. Med..

[35] Pal SK (2022). Adjuvant atezolizumab versus placebo for patients with renal cell carcinoma at increased risk of recurrence following resection (IMmotion010): a multicentre, randomised, double-blind, phase 3 trial. Lancet.

[36] Motzer RJ (2023). Adjuvant nivolumab plus ipilimumab versus placebo for localised renal cell carcinoma after nephrectomy (CheckMate 914): a double-blind, randomised, phase 3 trial. Lancet.

[37] Bigot P (2022). French AFU Cancer Committee Guidelines—Update 2022-2024: management of kidney cancer. Progrès. Urologie.

[38] Raghunathan, T., Lepkowski, J., Hoewyk, J. & Solenberger, P. A multivariate technique for multiply imputing missing values using a sequence of regression models. *Surv. Methodol.* **27** (2000).

[39] Azur MJ, Stuart EA, Frangakis C, Leaf PJ (2011). Multiple imputation by chained equations: what is it and how does it work?. Int. J. Methods Psychiatr. Res..

[40] Cox DR (1972). Regression models and life-tables. J. R. Stat. Soc.: Ser. B (Methodol.).

[41] Blanche P, Kattan MW, Gerds TA (2019). The c-index is not proper for the evaluation of t-year predicted risks. Biostatistics.

[42] Uno H, Cai T, Pencina MJ, D’Agostino RB, Wei LJ (2011). On the C-statistics for evaluating overall adequacy of risk prediction procedures with censored survival data. Stat. Med..

[43] Nadeau C, Bengio Y (2003). Inference for the generalization error. Mach. Learn..

[44] Lundberg SM (2020). From local explanations to global understanding with explainable AI for trees. Nat. Mach. Intell..

[45] Frank I (2002). An outcome prediction model for patients with clear cell renal cell carcinoma treated with radical nephrectomy based on tumor stage, size, grade and necrosis: the Ssign Score. J. Urol..

[46] Buti S (2017). Validation of a new prognostic model to easily predict outcome in renal cell carcinoma: the GRANT score applied to the ASSURE trial population. Ann. Oncol..

[47] Leibovich BC (2018). Predicting oncologic outcomes in renal cell carcinoma after surgery. Eur. Urol..

[48] Rivera SC (2020). Guidelines for clinical trial protocols for interventions involving artificial intelligence: the SPIRIT-AI extension. Lancet Digital Health.

