# Supplementary figures and images for: UroPredict: Machine learning model on real-world data for prediction of kidney cancer recurrence (UroCCR-120)
Source: NPJ Precis Oncol. 2024 Feb 23;8:45. doi: 10.1038/s41698-024-00532-x (PMC10891119; doi:10.1038/s41698-024-00532-x)

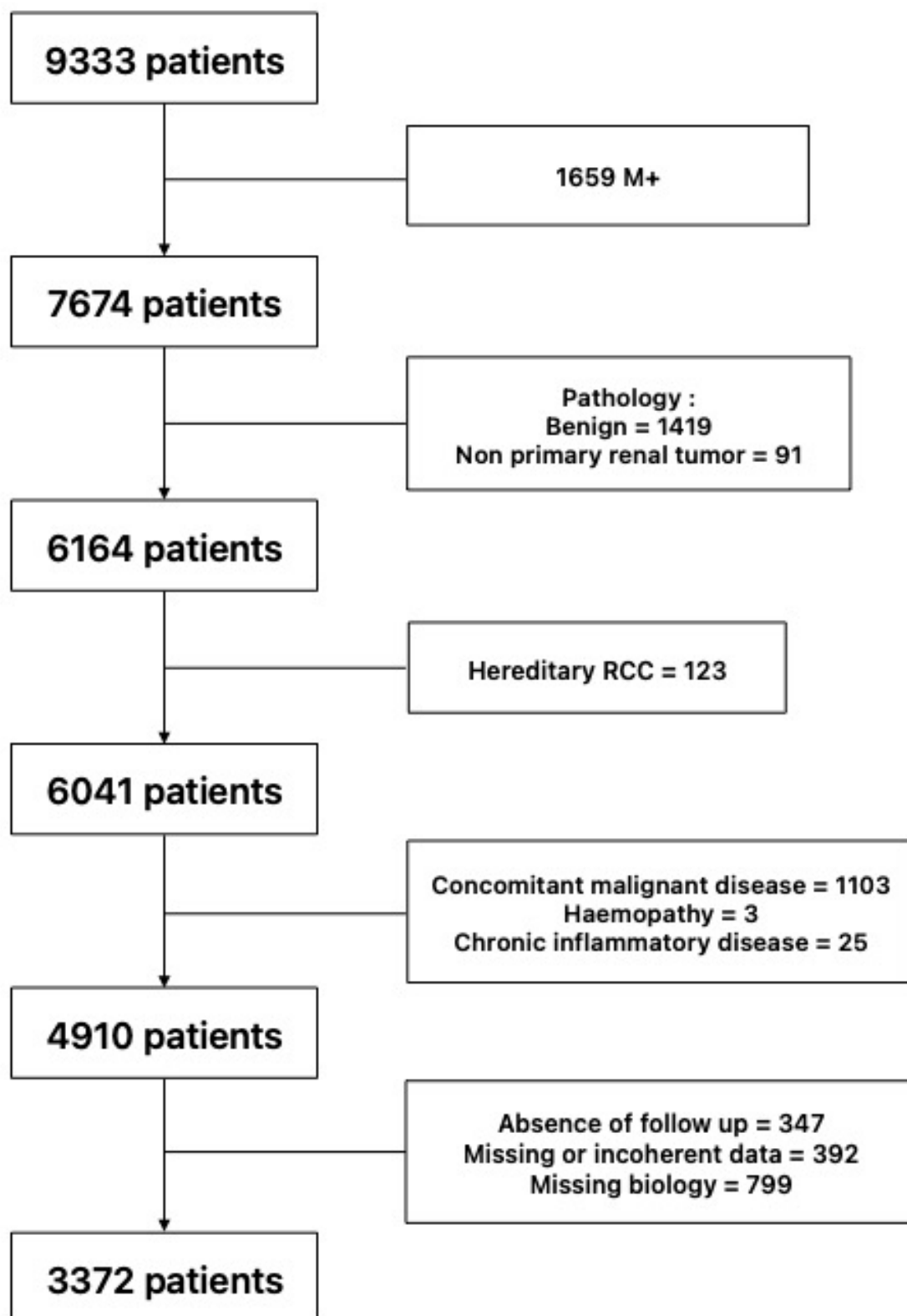

Supplementary figure 1. Flowchart of patient selection

Supplement: Supplementary file 1 — Supplementary figure 1 [file 41698_2024_532_MOESM1_ESM.pdf]
